# Introduction to the Site-specific Etiologic Results From the Pneumonia Etiology Research for Child Health (PERCH) Study

**DOI:** 10.1097/INF.0000000000002778

**Published:** 2021-08-25

**Authors:** Maria Deloria Knoll, Christine Prosperi, Henry C. Baggett, W. Abdullah Brooks, Daniel R. Feikin, Laura L. Hammitt, Stephen R.C. Howie, Karen L. Kotloff, Shabir A. Madhi, David R. Murdoch, J. Anthony G. Scott, Donald M. Thea, Katherine L. O’Brien

**Affiliations:** From the * Department of International Health, International Vaccine Access Center, Johns Hopkins Bloomberg School of Public Health, Baltimore, Maryland; † Division of Global Health Protection, Centers for Disease Control and Prevention, Atlanta, Georgia; ‡ Department of International Health, Johns Hopkins Bloomberg School of Public Health, Baltimore, Maryland; § International Centre for Diarrhoeal Disease Research, Bangladesh (icddr,b), Dhaka and Matlab, Bangladesh; ¶ Kenya Medical Research Institute—Wellcome Trust Research Programme, Kilifi, Kenya; ‖ Medical Research Council Unit The Gambia at London School of Hygiene & Tropical Medicine, Basse, The Gambia; ** Department of Paediatrics University of Auckland, New Zealand; †† Department of Pediatrics, Center for Vaccine Development and Global Health, University of Maryland School of Medicine, Baltimore, Maryland; ‡‡ Medical Research Council: Respiratory and Meningeal Pathogens Research Unit, University of the Witwatersrand, Johannesburg, South Africa; §§ Department of Science and Technology/National Research Foundation: Vaccine Preventable Diseases Unit, University of the Witwatersrand, Johannesburg, South Africa; ¶¶ Department of Pathology, University of Otago, Christchurch, New Zealand; ‖‖ Microbiology Unit, Canterbury Health Laboratories, Christchurch, New Zealand; *** Department of Infectious Disease Epidemiology, London School of Hygiene & Tropical Medicine, London, United Kingdom; ††† Department of Global Health and Development, Boston University School of Public Health, Boston, Massachusetts.

**Keywords:** pneumonia, etiology, childhood, PERCH

## Abstract

The Pneumonia Etiology Research for Child Health (PERCH) study evaluated the etiology of severe and very severe pneumonia in children hospitalized in 7 African and Asian countries. Here, we summarize the highlights of in-depth site-specific etiology analyses published separately in this issue, including how etiology varies by age, mortality status, malnutrition, severity, HIV status, and more. These site-specific results impart important lessons that can inform disease control policy implications.

The Pneumonia Etiology Research for Child Health (PERCH) study was the largest, most comprehensive pneumonia etiology study conducted in over 3 decades.^1^ Its aim was to estimate the etiologic fraction of pneumonia occurring in settings where child mortality from pneumonia is still far too high. This evidence could be used to guide clinicians, governments, donors and vaccine developers and to inform treatment and prevention strategies. Over 2 consecutive years at each site (between 2011 and 2014) in 7 countries in Africa and Asia (The Gambia, Mali, Zambia, South Africa, Kenya, Bangladesh and Thailand), the PERCH study team enrolled 4232 children 1–59 months of age who were hospitalized with severe pneumonia and 5325 control children from the same communities who did not have severe pneumonia. Children were tested for over 30 potential pathogens in multiple specimens, and using a Bayesian, partial latent class analysis, we estimated probabilities of etiologic agents (ie, etiologic fraction) at the individual and population level.^1^

The overall findings among HIV-uninfected children in PERCH, published previously, estimated that 61% of severe pneumonia cases were caused by viruses; respiratory syncytial virus A/B (RSV) alone accounted for 31% of cases.^1^ That publication included some, but limited, site-specific etiology results. Those results also excluded children living with HIV. Although viruses, especially RSV, were estimated to be the dominant cause at all 7 sites, the sites were heterogeneous in their settings and in the characterization of the children with severe/very severe pneumonia they enrolled (Tables 1 and 2). In this special issue, site-specific etiology was explored in more depth, stratified by factors such as age, mortality, malnutrition, severity, HIV status and more. Selected site-specific results contained in this special issue are highlighted in Table 2. Key findings across the 9 site-specific reports (2 each from Zambia and South Africa) include:

**TABLE 1.** Characteristics of PERCH Sites and Enrolled Cases

Country	Site	Urban/Rural	Time between Vaccine Introduction and PERCH Enrollment		Number of Cases Enrolled*	Characteristics of Enrolled Cases*						Page Nos.
						Age <1 Year, %	Very Severe Pneumonia, %†	Hypoxemia, %‡	Auscultatory Wheezing, %	CXR-Positive, %§	Case Fatality Ratio¶	
			Hib Vaccine	PCV								
The Gambia	Basse	Rural	15 years	2 years	631	63	15	7.8	32	47	4.3	S7–S17
Mali	Bamako	Urban	5 years	1 year	653	68	52	47	18	47	16	S18–S28
Kenya	Kilifi	Rural	10 years	6 months	630	54	51	30	13	50	5.9	S29–S39
Zambia	Lusaka	Urban	7 years	During PERCH (last 3 months)	HIV-uninfected: 514	78	31	36	12	57	16	S40–S49
					HIV-positive: 103	75	35	60	5.8	87	42	S50–S58
South Africa	Soweto	Urban	12 years	2.5 years	HIV-uninfected: 805	75	32	75	34	64	3.0	S59–S68
					HIV- positive: 115	72	34	78	15	88	19	S69–S78
Bangladesh	Dhaka	Urban	3 years	N/A‖	525	49	10	8.2	97	46	1.0	S79–S90
	Matlab	Rural										S79–S90
Thailand	Sa Kaeo,Nakhon Phanom	Rural/Periurban	N/A	N/A‖	223	39	23	24	46	50	4.0	S91–S100

*Restricted to HIV-uninfected cases at all sites except Zambia and South Africa where the number of HIV-uninfected and HIV-infected cases is reported separately.

†Very severe pneumonia defined as cough or difficulty breathing and at least 1 of the following signs: central cyanosis, difficulty breast-feeding or drinking, vomiting everything, convulsions, lethargy, unconsciousness or head nodding.

‡Hypoxemia defined as arterial oxygen saturation <92% on room air (<90% for sites at elevation: Zambia and South Africa) or a requirement for supplemental oxygen on admission if a room air reading was not available; a room air oxygen saturation reading was available for 3514 (88%) children; the South African site was at an altitude of 1600 meters above sea level and had a standard clinical practice to administer supplemental oxygen for all children admitted to hospital with a diagnosis of severe or very severe pneumonia.

§CXR positive defined as consolidation and/or other infiltrate. Restricted to interpretable chest radiographs.

¶Died in hospital or postdischarge but within 30 days of admission.

‖N/A: PCV not in use by national immunization program during PERCH study.

CXR indicates chest radiograph; N/A, not applicable; PERCH, Pneumonia Etiology Research for Child Health; Hib, *Haemophilus influenzae* type b vaccine; PCV, pneumococcal conjugate vaccine.

**TABLE 2.** Features of PERCH Study Sites and Highlights of Site-specific Findings

Site	Highlights
**The Gambia**^2^ *Setting:* Low GDP per capita, rural, a high child mortality rate, seasonal malaria transmission and a low HIV infection prevalence.	• Low proportion of cases were exposed to antibiotics before specimen collection (10%). • Amongst controls 1 year of age or older, 93% were fully vaccinated for age against *Haemophilus influenzae* and 80% against *S. pneumoniae*. • Positive blood cultures were observed in 14 (5%) of CXR-positive cases: 7 (50%) were *S. pneumoniae* (n = 2 PCV13 serotypes and n = 5 non-PCV13 serotypes), 2 were *H. influenzae* nontype b and the remaining were single occurrences of other pathogens. • Lung aspirates were available from 21 CXR-positive cases (most of any site) with *S. pneumoniae*, *Moraxella catarrhalis*, and *H. influenzae* being the most commonly detected pathogens. • Amongst CXR-positive cases, RSV (37%) and *S. pneumoniae* (13%) were the leading causes, followed by parainfluenza viruses (9.2%), *Mycobacterium tuberculosis* (8.3%) and *H. influenzae* (4.7%), with viruses accounting for 58% of cases and bacteria 28%. However, in CXR-positive very severe pneumonia cases, bacterial causes dominated (77%) with *S. pneumoniae* (41%) being the leading cause. • Epidemic pathogens such as parainfluenza viruses, notably Types 1 and 3, featured strongly.
**Mali**^3^ *Setting:* Low GDP per capita, high-density urban, high child mortality rate, seasonal malaria transmission, low HIV infection prevalence, well-established Hib vaccine program, PCV introduced <1 year before start of the study.	• The case fatality was high (13%) and etiology differed between fatal and nonfatal cases, with more bacteria and fungi among fatal cases: *Staphylococcus aureus*, *Pneumocystis jirovecii*, and *H. influenzae* type b together accounted for 39% of fatal cases compared with only 6.9% of those who survived, while RSV, HMPV and PIV-3 together comprised 6.5% of fatal cases compared with 48% of cases who survived. • Pneumonia in malnourished children was less frequently caused by RSV and HMPV compared with other children (combined 15% vs. 42% for weight-for-height and 7.2% vs. 40% for height-for-age), and instead was more commonly caused by bacteria. The top causes in malnourished children with weight-for-height Z scores below −2 SD were *H. influenzae* (21%), *S. aureus* (14%) and *S. pneumoniae* (12%). • Despite parental report that at least 60% of CXR+ cases were vaccinated with both PCV and Hib vaccines, most positive blood cultures (n = 12) were vaccine-preventable serotypes: 3 of 6 *S. pneumoniae* were PCV13-type (6B, 19A and 23F), 4 of 5 *H. influenzae* were type b, and 1 was positive for nontyphoidal *Salmonella spp*. • No culture isolates were recovered from 9 lung aspirates.
**Kenya**^4^ *Setting:* Predominantly rural, low HIV prevalence, mature Hib vaccine program and recent PCV program introduced with catch-up campaign strategies for all children under 5 years. Endemic malaria but transmission declining over past 15 years.	• Viruses accounted for 77% of the attribution of CXR-positive pneumonia. RSV was the main cause of CXR-positive pneumonia, followed by rhinovirus. The small contribution of Hib and pneumococcus to pneumonia may reflect the impact of vaccine introductions in this population. • Evaluated discharge diagnoses (*not done at other sites*): 28% of PERCH cases were not assigned LRTI in either their primary or secondary discharge diagnosis. The percentage without an LRTI diagnosis was higher for very severe pneumonia (42%) than for severe pneumonia (14%). Some (20%) of non-LRTI diagnosed cases had CXR abnormalities consistent with pneumonia. Some non-LRTI diagnoses included clinical presentations that can co-occur with pneumonia (e.g., malnutrition, malaria, anemia, febrile convulsions, pulmonary TB, immunosuppression, sepsis, sickle cell disease and meningitis).
**Zambia**^5,6^ *Setting:* Impoverished, densely populated, urban population with high HIV and *M. tuberculosis* prevalence and limited access to high-quality health care. Hib vaccine widely available but PCV not introduced until 3 months before the end of the study. Study conducted at a referral facility with most children receiving antibiotics before enrollment.	• Mortality among HIV-uninfected children was high (16%) and was higher among HIV-exposed (21%) compared with unexposed (11%) children. Outcomes were poor for HIV-infected children, with 40% dying in hospital. • Etiology among HIV-uninfected was highest for RSV (26%), *M. tuberculosis* (13%) and HMPV (13%). In contrast, for HIV-infected children, RSV contributed to only 3.7% of CXR-positive cases while bacterial and fungal pathogens accounted for over 75%: *S. pneumoniae* (20%), *S. aureus* (13%), *P. jirovecii* (25%) and *M. tuberculosis* (4.5%). • In Zambia, no difference in RSV etiologic fraction was seen by age. By contrast at all other sites, the proportion of pneumonia due to RSV in HIV-uninfected cases was higher in children <1 year of age. RSV contributed relatively little to the pneumonia mortality. • Among fatal HIV-uninfected cases, most (46%) were caused by *H. influenzae* type b, *S. aureus*, *P. jirovecii* and fungi, compared with 5.1% among cases that survived, while RSV, rhinovirus and HMPV caused only 3.6% of pneumonia in fatal cases compared with 47% among cases that survived. • Despite only a handful of children enrolled at the end of the study being fully immunized with PCV10 in Zambia, disease due to PCV10-type *S. pneumoniae* was rare (1.7%). However, lack of detection may in part be due to the near-universal practice of giving 1 dose of antibiotic before referral to the PERCH study facility—approximately 90% received antibiotics before admission. Despite this, among HIV-uninfected children, CXR-positive pneumonia was caused by treatable organisms in a large (38%) portion of cases. Potentially treatable causes of pneumonia accounted for 6 of the top 10 pathogens. • Pediatric *M. tuberculosis* is a significant cause of pneumonia for hospitalized children, both HIV-infected and uninfected. • Assessment of HIV-related care and treatment of HIV-infected cases occurring before admission revealed that only 35% had been receiving prophylactic co-trimoxazole, 14% had received anti-retroviral therapy and only 37% of caregivers knew their child’s HIV status at time of enrollment. • Fatal HIV-uninfected pneumonia cases were more likely to be malnourished (62% vs. 26%), be HIV-exposed (38% vs. 24%), sleep with 4 or more people in the same room (29% vs. 14%) and they waited longer before coming to the hospital (>5 days ill: 35% vs. 19%). Their pneumonia was more severe at admission: any danger sign (59% vs. 26%), lethargy (42% vs. 7.3%), hypoxia (60% vs. 32%) and many other clinical and laboratory differences.
**South Africa^7,8^** *Setting:* Low-middle income sub-Saharan African setting with established vaccination programs against Hib and pneumococcus as well as a high HIV and tuberculosis burden.	• Results are presented for both children living with and without HIV, including stratified by HIV-exposure status among HIV-uninfected children. Pneumonia etiology among children with HIV was proportionally less commonly attributed to viral pathogens (27%) than among HIV-exposed uninfected (50%) and HIV-unexposed (58%) children. Among children with HIV, *P. jirovecii*, *S. aureus*, *S. pneumoniae* and RSV were the most common causes; *P. jirovecii* and RSV featured almost exclusively amongst children <12 months. There was limited attribution to cytomegalovirus as a cause of pneumonia in the children with HIV. • Bacteremia (6.7%) and in-hospital death (10%) were frequent amongst children living with HIV who had radiologically confirmed disease. Clinical and laboratory characteristics of fatal cases are presented. • *M. tuberculosis* was commonly attributed (9.7%) as the cause of pneumonia in HIV-uninfected children. • The impact of sensitivity for detecting *M. tuberculosis* in a high burden setting is evaluated since more intensive screening was conducted at this site and the impact on etiologic conclusions is explored.
**Bangladesh**^9^ *Setting:* Low HIV-seroprevalence and low seasonal malaria transmission. Hib introduced in 2009. PCV introduced after PERCH. Few children exposed to antibiotics before specimen collection.	• The clinical manifestations of the pneumonia syndrome were different from that at the other sites, with the majority of cases having crackles (93%) and wheeze (97%), and very few with very severe pneumonia (11%). • Only 3 cases were blood culture positive, all *Enterobacteriaceae*, despite only 20% with evidence of antibiotics before admission. Although no cases were blood culture positive for *Streptococcus pneumoniae*, positives were detected at normal background rates among PERCH-ineligible children during the same time period. • No organisms were recovered from the 4 lung aspirates collected. • Detection of influenza was rare in PERCH cases and controls; surveillance systems operating during PERCH confirmed no substantial circulation of influenza was detected. • Most etiology was attributed to viruses (78%), driven primarily by RSV (31%) and rhinovirus (23%). There was limited evidence for influenza or *S. pneumoniae*. • Only minor differences were seen between urban and rural settings.
**Thailand**^10^ *Setting:* Middle income setting, no Hib or PCV use	• RSV and *M. tuberculosis* accounted for 35% and 10%, respectively, of the total etiologic fraction of CXR-positive cases. • No pneumococci were identified on culture despite lack of PCV use in the national immunization program. Other research shows invasive pneumococcal disease is not uncommon in Thailand. It may not have been detected in the PERCH study due to prior treatment with antibiotics, either because antibiotics reduce culture sensitivity or because they prevent progression to severe pneumonia. • Comorbidities, primarily developmental delay, were an important contributing factor to fatalities.

Hib indicates *Haemophilus influenzae* type b vaccine; PCV, pneumococcal conjugate vaccine; PERCH, Pneumonia Etiology Research for Child Health; RSV, respiratory syncytial virus; CXR, Chest radiograph; LRTI, Lower respiratory tract infection; HMPV, human metapneumovirus; PIV-3, parainfluenza virus type 3.

- *Etiologic results for the full list of pathogens at each site:* Previously published PERCH results described site-specific etiology caused by 10 of the over 30 pathogens evaluated; for some sites, such as Thailand,^10^ those pathogens accounted for only about half of the attributed etiology.
- *Effects of age and severity are examined in more depth:* Differences are revealed between sites; for example, the etiologic fraction of pneumonia attributed to RSV was higher in children <1 year than 1–4 years at all sites except Zambia^5^ where the etiologic fraction was similarly high in both age groups.
- *Etiology of pneumonia in children living with HIV are shown for the first time*: Two sites, South Africa^8^ and Zambia,^6^ had relatively high prevalence of HIV in their hospitalized cases (12% and 17%). Bacteremia and in-hospital death were frequent among children with HIV at both sites. Bacterial and fungal pathogens, including *Pneumocystis jirovecii*, *Streptococcus pneumoniae*, *Staphylococcus aureus*, contributed a considerable burden of radiologically confirmed pneumonia in cases at both sites.
- *Etiology of pneumonia in HIV-exposed but uninfected (HEU*): Children whose mothers were living with HIV but who were themselves uninfected are examined in South Africa^7^ and Zambia^5^ and compared with children whose mothers did not have HIV. In Zambia, mortality among HEU cases was nearly double that of HIV-unexposed cases. Etiology results differed between the 2 sites: in South Africa, the etiology was similar between HEU- and HIV-unexposed pneumonia cases, while in Zambia, etiology among HEU cases shared features of children with HIV and unexposed.
- *Etiology by mortality status*: While RSV was the most common cause of severe pneumonia overall, bacteria and fungi were more common than RSV as the cause among cases who died. Mali^3^ and Zambia^5^ had the highest case fatality ratios among PERCH sites and were a focus of etiology evaluation among the fatal cases. In Mali, *S. aureus*, *P. jirovecii* and *Haemophilus influenzae* type b together accounted for 40% of fatal cases (compared with only 6.9% of those who survived), while RSV, human metapneumovirus (HMPV) and parainfluenza virus (PIV) type 3 together comprised 6.5% (compared with 48% of cases who survived). In Zambia, most (46%) fatal cases were caused by *H. influenzae* type b, *S. aureus*, *P. jirovecii* and other fungi, compared with 5.1% among cases that survived, while RSV, rhinovirus and HMPV caused 3.6% of pneumonia in fatal cases compared with 47% among cases that survived.
- *Clinical characteristics associated with mortality*: In Zambia,^5^ fatal pneumonia cases were more likely to have a longer illness duration before admission, more severe disease, HIV exposure and to be malnourished.
- *Role of comorbidities in mortality*: The frequency and influence of comorbidities in cases from Thailand^10^ was explored and found to be comparable with that at the other PERCH sites. Comorbidities were an important contributing factor to fatalities at all sites. In Thailand, 78% of fatal cases had a comorbidity, primarily a developmental delay; comorbidities were present in 44% of cases that died in The Gambia^2^ and Zambia^5^ and in 67%–80% of those who died in Mali,^3^ Kenya^4^ and Bangladesh.^9^ In Mali, moderate to severe wasting and stunting were associated with an increased likelihood of a bacterial etiology and increased the likelihood of a fatal outcome.
- *Role of malnutrition on etiology*: Pneumonia in severely malnourished cases, explored in children from Mali,^3^ was proportionally much less frequently attributed to RSV and HMPV compared with other children (combined 15% vs. 42% for weight-for-height <−2 SD and 7.2% vs. 40% for height-for-age Z scores <−2 SD). The probability of *H. influenzae* (21% vs. 2.9%), *S. aureus* (14% vs. 2.6%) and *Mycobacterium tuberculosis* (6.9% vs. 0.5%) was substantially more common in cases with low weight-for-height Z scores than in cases with higher Z scores. The etiology of pneumonia in severely malnourished cases was more evenly distributed across pathogens but perhaps with more bacterial than viral pathogens as the most common causes; for children with height-for-age Z scores <−2 SD, the leading causes were *S. pneumoniae* (11%) and *H. influenzae* (9.7%).
- *Findings from lung aspirates are compared with pre-Hib vaccine and pre-PCV time periods*: In The Gambia^2^ where there was substantial prior experience collecting lung aspirate samples, *H. influenzae* type b (n = 1) was seen less during PERCH compared with earlier studies but PCV-type pneumococcus was still observed during this early PCV use period (PCV was introduced 2 years prior to PERCH), reinforcing the need for ongoing monitoring through surveillance. *S. pneumoniae*, *Moraxella catarrhalis* and *H. influenzae* were the only pathogens detected in lung aspirates of Gambian cases.
- *PERCH severe pneumonia etiology is considered in the context of surveillance data*: The magnitude of severe pneumonia attributed to influenza and pneumococcus was smaller than expected. PERCH did not find evidence that influenza virus caused a large portion of the pneumonias during the study period. Local surveillance data on influenza and other seasonal pathogens support the low influenza prevalence in cases and controls during the period of the PERCH study, notwithstanding that some sites have reported pneumonia associated with influenza in periods before and after the PERCH study. Bangladesh^9^ and Thailand^10^ did not detect any pneumococcal positive blood cultures during PERCH, but studies of less severe respiratory illness and bloodstream infections at those sites demonstrate that pneumococcal disease was present during the PERCH enrollment period. Potential explanations are explored for the lack of pneumococcal disease detection in PERCH cases in these settings where pneumococcal conjugate vaccine was not in use.
- *Specificity of the PERCH severe and very severe pneumonia case definition:* PERCH used the WHO-defined severe or very severe pneumonia case definition, which is designed to favor sensitivity at the expense of specificity. The Kenya^4^ site explored the specificity of the case definition by examining the primary hospital-assigned discharge diagnoses among all admissions and among PERCH cases. Lower respiratory tract infection (LRTI) was recorded more often as a primary discharge diagnosis in PERCH enrollees (66%) than in all admissions (25%), but 28% of PERCH cases were not assigned LRTI in either primary or secondary discharge diagnoses. The proportion of cases without LRTI listed as a discharge diagnosis was higher for those with PERCH-defined very severe pneumonia (42%) than for severe pneumonia (14%). However, hospital-assigned diagnosis is not a gold standard, as shown by the fact that 15% of CXR-positive cases did not have a discharge diagnosis of LRTI.
- *Impact of repeat M. tuberculosis testing in a high prevalence setting*: Assumptions were required in the PERCH etiology analysis regarding the sensitivity for all pathogen/specimen tests conducted, including for *M. tuberculosis*. South Africa^7^ performed multiple *M. tuberculosis* tests on individual cases over several days while other sites tested only once, on the day of admission. The South Africa site reestimated the fraction of pneumonia attributed to *M. tuberculosis* using any positive test and increased sensitivity to account for this from the range of 10%–30% to 20%–50%. The result was a change in the estimated proportion attributed to *M. tuberculosis* from 6.4% to 9.7%.

This collection of results is part of a suite of papers^1,11^ and should be taken in that context. The results represent sites with diverse epidemiologic settings, including 2 geographic regions, differences in altitude, a range of low- and middle-income economic backgrounds, urban and rural communities, variations in HIV and malaria prevalence and variations in PCV and Hib vaccine use. Each of these factors likely contributes to variations in the etiologic spectrum and mortality of pneumonia in children. While a study like PERCH can provide important insights, it does not take the place of routine ongoing surveillance, which should continue, especially following new vaccine introduction. Equally important, our data are population data and cannot be extrapolated to the individual patient as an etiologic probability diagnosis. Particularly for the viruses, those that appear to be of greatest public health importance will likely require improved rapid or bedside diagnostics for individual patient diagnosis, as well as routine monitoring for prioritization for future therapeutic or preventive measures. Finally, PERCH was a study of severe and very severe childhood pneumonia, but its findings have relevance to all grades of childhood pneumonia severity in that, irrespective of primary etiology, given the prominence of bacterial infection in mortality, appropriate antimicrobial therapy early in infection likely remains important in reducing the overall pneumonia case fatality rate, and should continue.

In conclusion, these site-specific results impart 5 important lessons that have disease control policy implications.

1. Even though bacteria still account for many childhood pneumonia deaths, bacteria no longer account for the majority of pneumonia deaths as in past decades; most cases of severe and very severe pneumonia are viral, and thus primary prevention policies could be adapted accordingly.
2. A corollary to the point above is that many childhood pneumonia cases involve codetection of multiple pathogens, which could indicate coinfection amongst 2 or more of these. Our statistical model required single pathogen etiologic assignment to create a pie chart of all identified infecting agents. It was not intended to rule in or out coinfection. Multiple studies using both animal models and human epidemiologic data have identified coinfection, notably sequential viral–bacterial coinfection, as a contributor to very severe pneumonia and pneumonia mortality. The role of co-detected pathogens, whether one or both caused the pneumonia, deserves further investigation using a different study design than was used in PERCH.
3. Although the leading pathogens were common to all sites, we observed substantial variation in etiologic fraction by site, even on the same continent, which will affect disease control priorities, policies and even clinical practices. To further reduce child pneumonia mortality, control policies will need local/regional adaptation.
4. Within countries, there may be differences in relative pathogen prevalence between urban and rural settings that could affect clinical management, and therefore health policies and services may need to be correspondingly adapted. For example, HIV prevalence, and therefore *M. tuberculosis* and *P. jirovecii* prevalence, may be higher in population-dense urban settings than in rural settings, and therefore the etiologic fraction of all pneumonia due to these pathogens would be higher in urban versus rural health facilities. Local data will assist in adjusting health policy and practices accordingly.
5. Young age and comorbidities including malnutrition, small for gestational age and HIV-infection and exposure, increase the likelihood of mortality from childhood pneumonia and affect the fraction attributed to bacteria—epidemiologic findings that can inform disease control policies and practices.

We would like to thank the communities that voluntarily participated in these studies, not simply for their cooperation, but for the knowledge that they have contributed, which will benefit children worldwide, many not yet born, for years to come.

## ACKNOWLEDGMENTS

We offer sincere thanks to the study participants and their families. We recognize the efforts of the following groups during the study development, conduct, and analysis phases: Pneumonia Methods Working Group, Pneumonia Etiology Research for Child Health (PERCH) Expert Group, PERCH contributors and the PERCH Chest Radiograph Reading Panel. We also acknowledge the substantial contributions of all members of the PERCH Study Group (see below): PERCH Study Group. Johns Hopkins Bloomberg School of Public Health, Baltimore, Maryland: Katherine L. O’Brien (PI), Orin S. Levine (Former PI, current affiliation Bill & Melinda Gates Foundation, Seattle, Washington), Maria Deloria Knoll (co-PI), Daniel R. Feikin (joint affiliation with Centers for Disease Control and Prevention, Atlanta, Georgia), Andrea N. DeLuca, Amanda J. Driscoll, Nicholas Fancourt, Wei Fu, Meredith Haddix, Laura L. Hammitt, Melissa M. Higdon, E. Wangeci Kagucia, Ruth A. Karron, Mengying Li, Daniel E. Park, Christine Prosperi, Qiyuan Shi, Zhenke Wu, Scott L. Zeger; The Emmes Corporation, Rockville, Maryland: Nora L. Watson; Nuffield Department of Clinical Medicine, University of Oxford, United Kingdom: Jane Crawley; University of Otago, Christchurch, New Zealand: David R. Murdoch; ICDDR, b, Dhaka and Matlab, Bangladesh: W. Abdullah Brooks (site PI), Hubert P. Endtz, Khalequ Zaman, Doli Goswami, Lokman Hossain, Yasmin Jahan, Mohammod Jobayer Chisti; Medical Research Council Unit The Gambia at London School of Hygiene & Tropical Medicine: Stephen R. C. Howie (site PI), Bernard E. Ebruke, Martin Antonio, Jessica McLellan, Eunice Machuka, Arifin Shamsul, Syed M.A. Zaman, Grant Mackenzie; KEMRI-Wellcome Trust Research Programme, Kilifi, Kenya: J. Anthony G. Scott (site PI and PERCH co-PI, joint affiliation with London School of Hygiene and Tropical Medicine, London, United Kingdom), Juliet O. Awori, Susan C. Morpeth, Alice Kamau, Sidi Kazungu, Micah Silaba Ominde; Department of Pediatrics and Department of Medicine, Center for Vaccine Development and Global Health, University of Maryland School of Medicine, Baltimore, Maryland and Centre pour le Développement des Vaccins (CVD-Mali), Bamako, Mali: Karen L. Kotloff (site PI), Milagritos D. Tapia, Samba O. Sow (site co-PI), Mamadou Sylla, Boubou Tamboura, Uma Onwuchekwa, Nana Kourouma, Aliou Toure, Seydou Sissoko; Medical Research Council: Respiratory and Meningeal Pathogens Research Unit and Department of Science and Technology/National Research Foundation: Vaccine Preventable Diseases, University of the Witwatersrand, Johannesburg, South Africa: Shabir A. Madhi (site PI), David P. Moore, Peter V. Adrian, Vicky L. Baillie, Locadiah Kuwanda, Azwifarwi Mudau, Michelle J. Groome, Nasreen Mahomed, Eric A.F. Simões; Thailand Ministry of Public Health—U.S. CDC Collaboration, Nonthaburi, Thailand: Henry C. Baggett (site PI), Somsak Thamthitiwat, Susan A. Maloney (former site PI), Charatdao Bunthi, Julia Rhodes, Pongpun Sawatwong, Pasakorn Akarasewi (site co-PI, Ministry of Public Health); Department of Global Health and Development, Boston University School of Public Health, Boston, Massachusetts and University Teaching Hospital, Lusaka, Zambia: Donald M. Thea (site PI), Lawrence Mwananyanda (site co-PI), James Chipeta (site co-PI), Phil Seidenberg (site co-PI), James Mwansa, Somwe wa Somwe, Geoffrey Kwenda; Canterbury Health Laboratories, Christchurch, New Zealand: Trevor P. Anderson, Joanne Mitchell, Shalika Jayawardena, Rose Watt.

This paper is published with the approval of the Director, Kenya Medical Research Institute.
